# Unlocking the Glioblastoma Enigma: Exploring PD-L1 (Programmed Death-Ligand 1) and IDH1 (Isocitrate Dehydrogenase-1) Expression and Their Immunotherapeutic Implications

**DOI:** 10.7759/cureus.76920

**Published:** 2025-01-04

**Authors:** Syeda Iqra Mushir, Summaya S Chaudry, Henna Azmat, Areeba Masood, Momina Habib, Ahmareen K Sheikh

**Affiliations:** 1 Histopathology, Shaheed Zulfiqar Ali Bhutto Medical University/Pakistan Institute of Medical Sciences (PIMS), Islamabad, PAK; 2 Pathology, Federal Government Polyclinic, Islamabad, PAK

**Keywords:** bevacizumab, glioblastoma multiforme (gbm), glioma, idh1 mutation, immune microenvironment, immunohistochemistry, immunotherapy, nivolumab, pd-l1 expression, tumor proportion score (tps)

## Abstract

Objective

In order to establish a connection between programmed death-ligand 1 (PD-L1) expression and glioma grades as well as the presence of IDH1 mutations, it is necessary to investigate the expression of PD-L1 and isocitrate dehydrogenase-1 (IDH1) in glioma patients and assess their potential as predictive markers for glioblastoma multiforme (GBM) immunotherapy. We analyzed the frequency of PD-L1 expression in glioma samples.

Methodology

In this two-year retrospective study, 45 glioma cases of varying grades (grades 2 to 4) were examined at a tertiary care hospital. Tumor samples that were formalin-fixed, paraffin-embedded (FFPE) were obtained from the pathology archives of the hospital. According to the WHO Classification of Central Nervous System Tumors, 5th edition, tumor grading and histopathological subtyping were carried out. PD-L1 antibody (clone 28-8) and IDH1 (R132H, clone QM002, Quartett Immunodiagnostika, 1:100 dilution) markers were used for immunohistochemistry (IHC). The sections underwent deparaffinization, rehydration, and antigen retrieval using Leica bond III staining platform. Based on the tumor proportion score (TPS), which is the proportion of viable tumor cells with membranous staining, PD-L1 expression was assessed. The literature's standardized cut-off values were used to determine positive expression. Staining intensity and tumor cell location were used to determine the status of IDH1 mutations. Age, sex, and tumor location were among the clinical and demographic information gathered about the patient. The association between PD-L1 expression, glioma grades, and IDH1 (R132H) mutation status was assessed statistically using SPSS software and a Chi-square test. The threshold for statistical significance was p < 0.05. For every IHC run, positive and negative controls were used as part of the quality control procedures. To reduce bias and guarantee consistency, two pathologists and post graduate residents independently reviewed the results.

Results

PD-L1 expression was found in 27 out of 36 (75%) grade 4 glioblastoma multiforme cases and six out of nine (66.7%) grade 2 gliomas. Overall, 33/45 (73.3%) of the gliomas had PD-L1 expression. However, PD-L1 expression and glioma grade did not correlate in a statistically significant way. IDH1 (R132H) expression and PD-L1 were found to be inversely correlated (p < 0.05).

Conclusion

The findings suggest that PD-L1 may be a promising therapeutic target, even in the absence of significant grade-specific trends by demonstrating PD-L1 presence in the majority of glioma cases, highlighting its potential as a therapeutic target in GBM immunotherapy. The results provide insight into the immune landscape of gliomas and pave the way for future research into effective combination therapies for GBM, despite the lack of a significant correlation between glioma grade and PD-L1 expression.

## Introduction

Nearly 49% of all cancers of the central nervous system are glioblastoma multiforme (GBM), the most aggressive and deadly primary brain tumor. With a median survival time of 12-15 months and a five-year survival rate of less than 10%, GBM still presents substantial treatment and prognostic challenges despite advancements in therapeutic modalities [1,2]. The tumor's highly infiltrative nature, molecular heterogeneity, resistance to traditional therapies, and the inability to achieve complete surgical resection due to infiltration into critical brain structures are some of the factors contributing to this bleak outlook [3,4].

GBM is usually treated with maximally safe surgical resection, radiotherapy, and concurrent chemotherapy with temozolomide. Although survival has been slightly increased by this regimen, recurrence and the emergence of therapeutic resistance frequently make it difficult to maintain. The tumor's persistence and progression are exacerbated by the blood-brain barrier (BBB), which also restricts the delivery of systemic treatments [5]. These restrictions highlight the urgent need for creative treatment approaches that can get past these barriers and improve patient outcomes.

By using the immune system to target tumor cells, recent developments in immunotherapy have demonstrated great promise in treating a variety of cancers. By attaching to the T cell's programmed death-1 (PD-1) receptor and inhibiting its activity, programmed death-ligand 1 (PD-L1), a crucial immune checkpoint protein, aids in tumor immune evasion and increases tumor survival [6,7]. Although PD-1/PD-L1 axis immune checkpoint inhibitors have transformed cancer treatment, their function in gliomas is still being studied. Although PD-L1 expression in glioblastoma has been recognized as a possible therapeutic target, more research is needed to fully understand its relationship to tumor grade and molecular features like isocitrate dehydrogenase (IDH1 R132H) mutations [8,9].

Lower-grade gliomas are more likely to have IDH1 (R132H) mutations, which are linked to improved prognosis and greater sensitivity to therapies like radiation and temozolomide [10,11]. The oncometabolite D-2-hydroxyglutarate (D-2HG), which is produced by these mutations, affects immunological responses, epigenetic reprogramming, and tumor progression. It is interesting to note how IDH1 (R132H) mutations and PD-L1 expression interact, pointing to a complicated relationship that could affect immune regulation and the tumor microenvironment (TME). The TME in glioblastoma is a dynamic and diverse ecosystem made up of immune cells, glioma cells, and an extracellular matrix. These elements work together to promote tumor growth, resistance to treatment, and immune evasion [12,13].

This study builds on previous research by examining the immune landscape in gliomas and examining the relationship among IDH1 (R132H) mutations, glioma grades, and PD-L1 expression. Assessing these markers using immunohistochemistry provides a way to find reliable biomarkers that may direct tailored immunotherapy strategies and enhance therapeutic results. In order to create targeted therapies that not only address the aggressive behavior of the tumor but also maximize immune responses to combat tumor immune evasion mechanisms, it is essential to comprehend these relationships.

The main goal of this study is to better understand how PD-L1 is expressed and interacts with IDH1 (R132H) within the glioblastoma tumor environment. By exploring this relationship, we aim to provide new insights into how glioblastomas evade the immune system. This could help identify potential targets for future treatments, addressing gaps in current knowledge and contributing to the development of more effective therapies. The knowledge acquired may help change the way that glioblastoma is treated from a one-size-fits-all strategy to a more customized and efficient framework, which would ultimately increase the survival and quality of life for those who are suffering from this terrible illness.

## Materials and methods

Study design and case selection

A total of 45 glioma cases were gathered from a tertiary care hospital's pathology archives between January 2020 and December 2022 for this retrospective, observational study. Thirty-six (80%) of these were high-grade gliomas (WHO Grades 3 and 4), while nine (20%) were low-grade gliomas (WHO Grade 2). All cases had to be primary gliomas, histologically confirmed by board-certified pathologists, and free of previous therapeutic interventions (such as chemotherapy, radiation, or immunotherapy) prior to tissue sample collection in order to meet the inclusion criteria. Cases representing recurrent tumors or those with inadequate clinical or pathological information were not included.

Tissue blocks from each included case that were formalin-fixed, paraffin-embedded (FFPE) were taken out for immunohistochemical examination. Sections that were each 4 µm thick were prepared and placed on slides that were positively charged. Following deparaffinization in xylene and rehydration with graded alcohols, antigen retrieval was performed [14].

At pre-validated dilutions, primary antibodies were applied against PD-L1 (clone 28-8) and IDH1 (R132H, clone QM002, Quartett Immunodiagnostika, 1:100 dilution) using the Leica Bond III staining platform. Following incubation, sections were visualized using diaminobenzidine (DAB) as the chromogen and treated with a polymer-based secondary antibody detection system. The counterstain used was hematoxylin. To guarantee assay specificity and reproducibility, suitable positive and negative control sections were incorporated into every staining run.

The IHC slides were independently assessed by two skilled pathologists and post graduate residents who were blind to all clinical information. Joint review was used to settle disagreements. Tumor cell membranous staining was used to measure PD-L1 expression, and cases were deemed positive if at least 1% of the tumor cells showed membranous staining, which corresponds to a tumor proportion score (TPS) of at least 1% [15]. The determination of IDH1 (R132H) positivity involved examining the tumor cells' nuclear and cytoplasmic immunoreactivity. IDH1-mutant cases were those that showed this staining, whereas IDH1-wildtype cases did not.

Data collection and statistical analysis

Patient demographics, tumor grade, IDH1 mutation status, and PD-L1 positivity were all documented. The distribution of each variable within the population under study was summarized using descriptive statistics, such as frequencies and percentages (n (%)). Pearson's correlation test was used to evaluate the relationship between PD-L1 and IDH1 (R132H) expression. A p-value of less than 0.05 was deemed statistically significant, and SPSS version 25 (IBM) was used for all statistical analyses.

Ethical considerations

This study was authorized by the tertiary care hospital's Institutional Review Board (IRB) and carried out in accordance with ethical guidelines. Because the study was retrospective in nature, informed consent was waived. To ensure confidentiality, all patient data and samples were completely anonymized.

## Results

**Figure 1:**
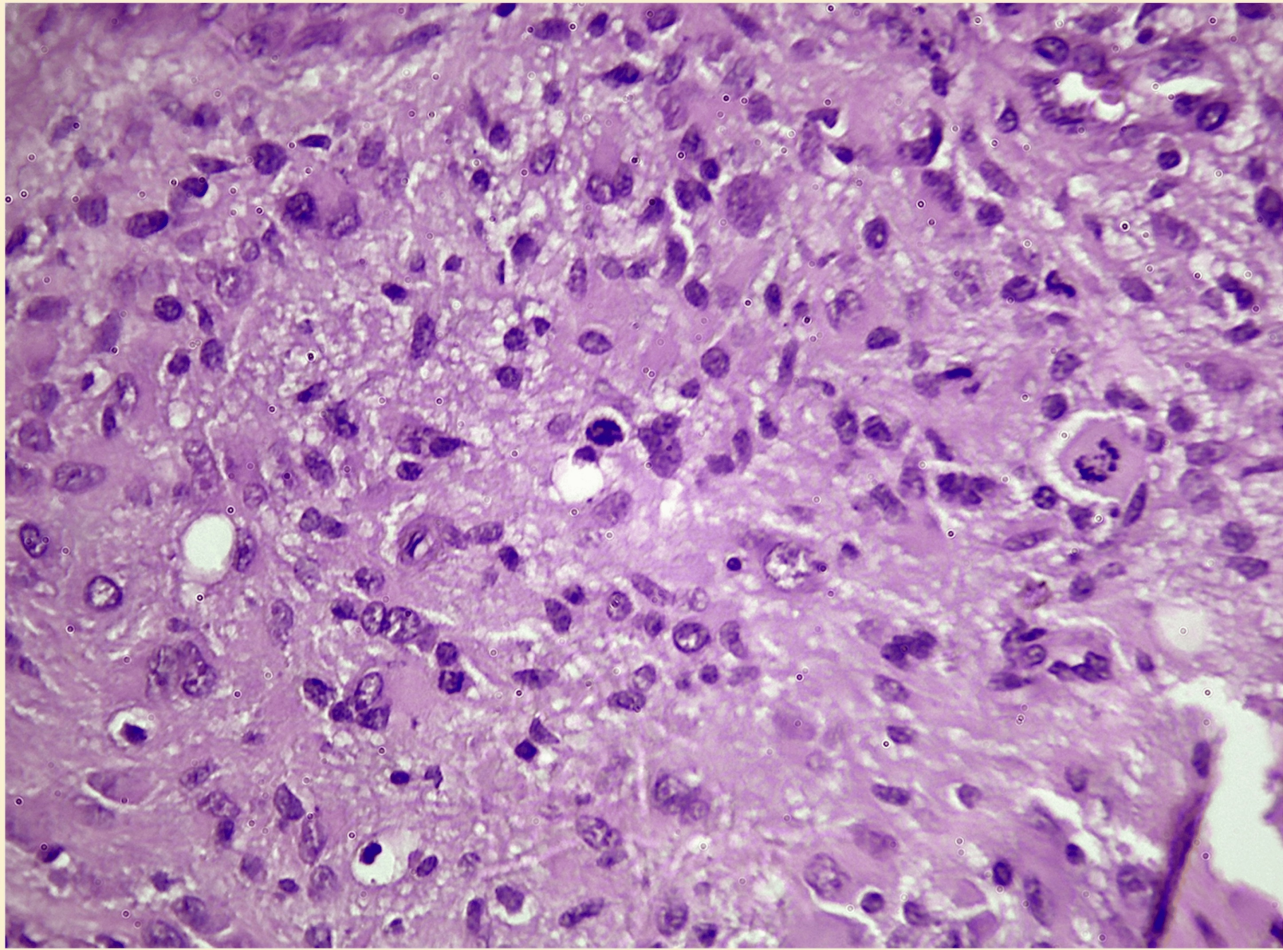
A typical hematoxylin and eosin (H&E)-stained section of a high-grade glioma (such as grade 4 glioblastoma) shows prominent nuclear atypia, frequent mitotic figures, and noticeably increased cellularity. The characteristic pseudopalisading necrosis is a result of the frequent presence of vascular proliferation and necrotic areas. These characteristics highlight how aggressive and highly invasive high-grade gliomas are.

**Figure 2:**
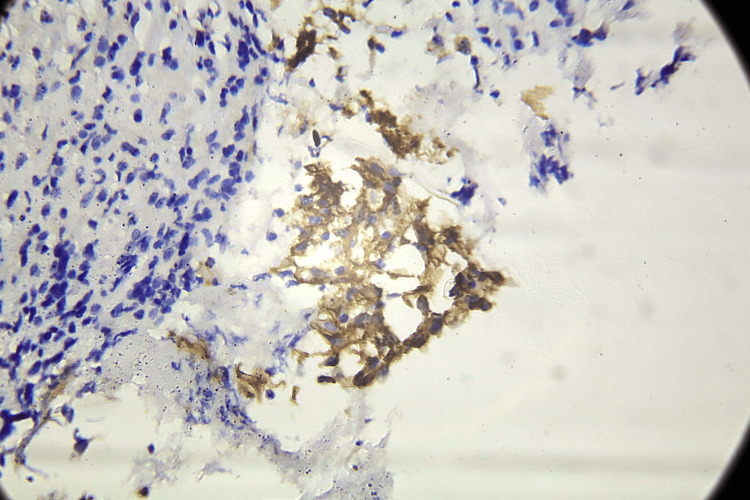
Strong membranous positivity is demonstrated by a representative section of a high-grade glioma stained for PD-L1. Strong, localized PD-L1 expression on the surface of tumor cells suggests a potentially immunosuppressive milieu. PD-L1: programmed death-ligand 1.

**Figure 3:**
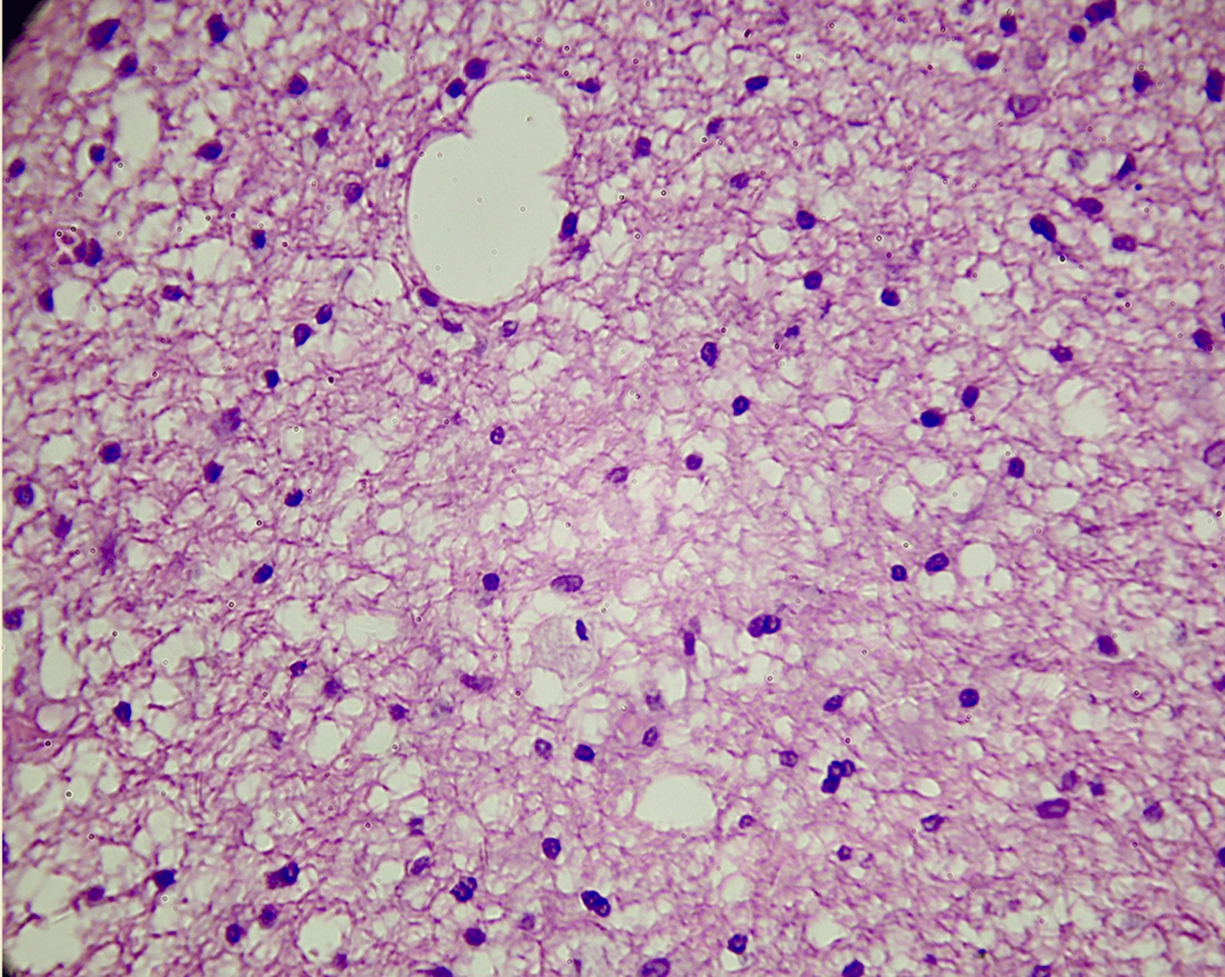
A representative section of a low-grade glioma (such as grade 2) stained with hematoxylin and eosin (H&E) reveals a more ordered architecture and comparatively lower cellular density than high-grade gliomas. The tumor cells exhibit reduced vascular proliferation, limited mitotic figures, and fewer pleomorphic nuclei. There are fewer signs of aggressive growth and a more consistent overall appearance.

**Figure 4:**
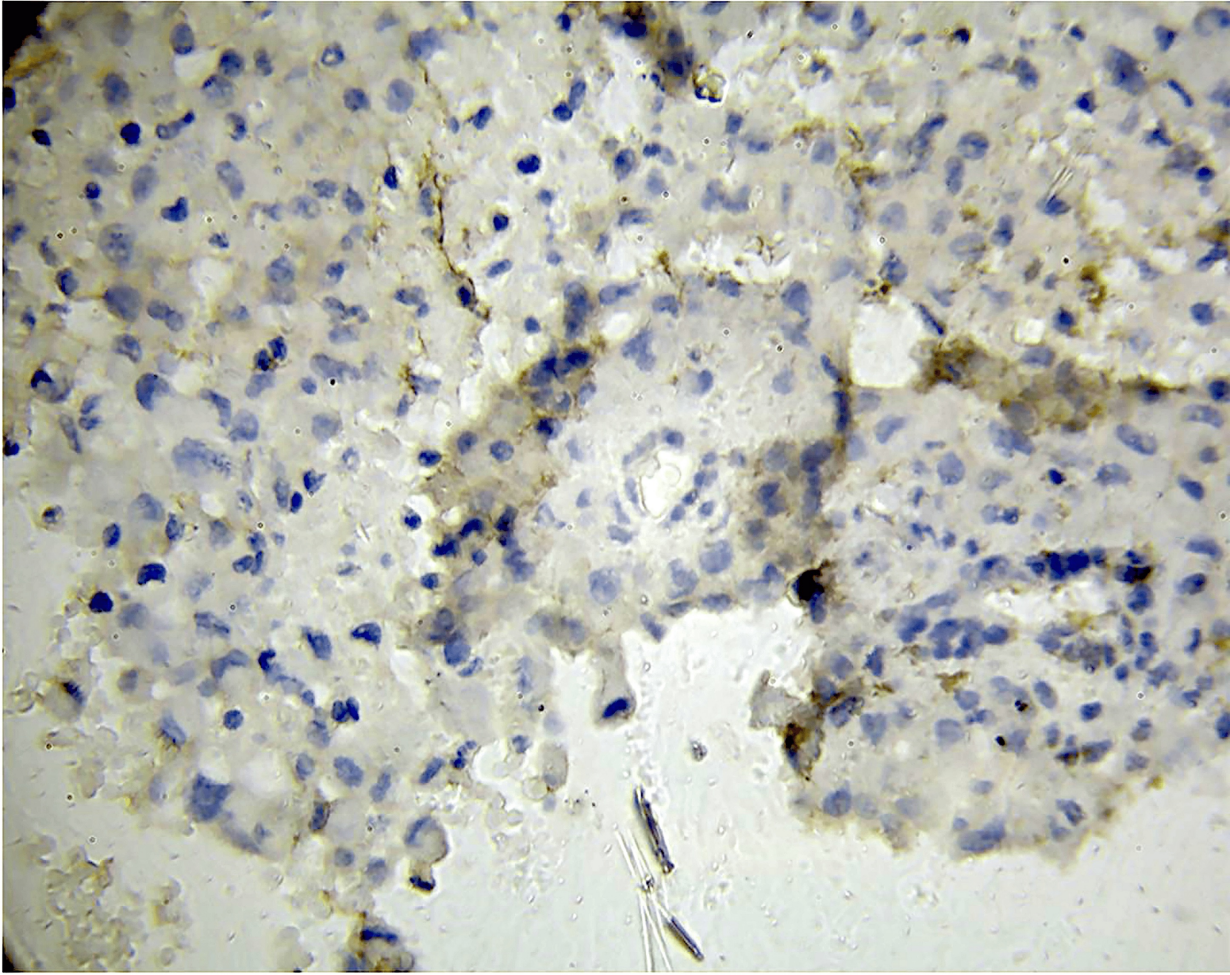
A subset of tumor cells exhibit mild to moderate membranous staining in a representative low-grade glioma section stained for PD-L1. This degree of PD-L1 expression indicates that even lower-grade gliomas may use some level of immune regulation, even though it is not as extensive or intense as in high-grade tumors. PD-L1: programmed death-ligand 1.

**Figure 5:**
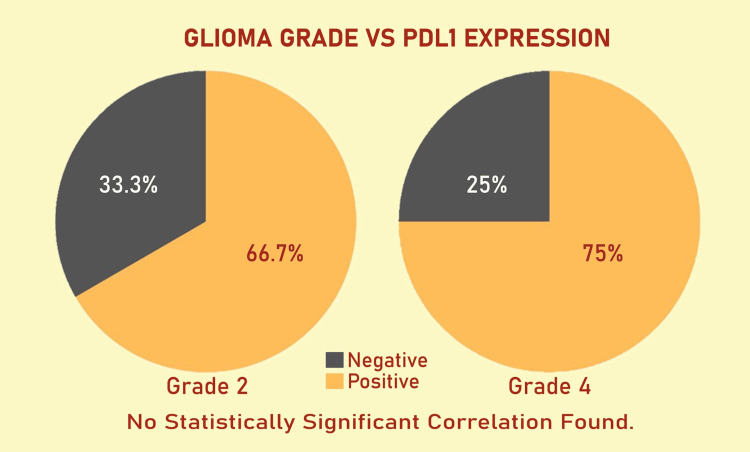
The percentage of PD-L1-positive cases in low-grade and high-grade gliomas is contrasted in two different pie charts. The percentage of tumors with membranous PD-L1 staining of ≥1% (PD-L1 positive) versus those with staining below this threshold (PD-L1 negative) is displayed in each chart. Low-grade gliomas are the subject of the first pie chart, which shows their comparatively lower but still significant PD-L1 positivity rate. The second pie chart illustrates how high-grade gliomas have a higher frequency of PD-L1 positivity. PD-L1: programmed death-ligand 1.

**Figure 6:**
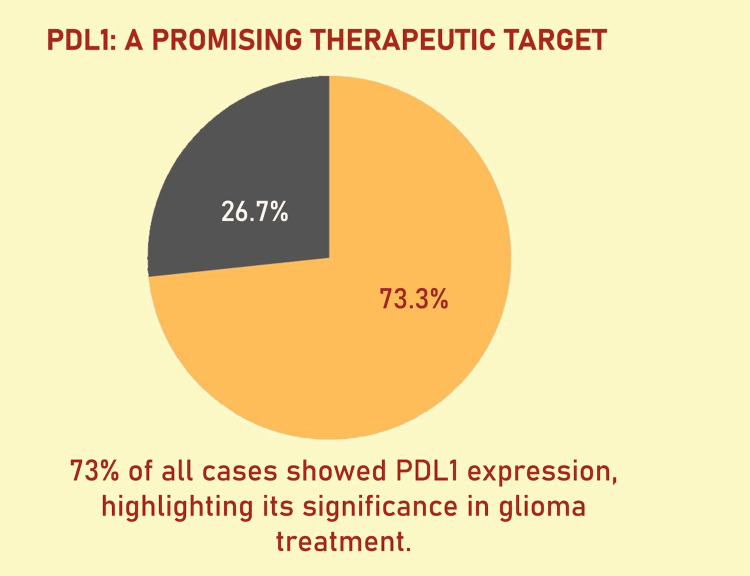
The overall percentage of glioma cases that tested positive for PD-L1 expression is shown in a pie chart. The fraction of cases that are PD-L1 positive (≥1% membranous staining) as opposed to PD-L1 negative is represented by the segments of the chart. PD-L1: programmed death-ligand 1.

**Figure 7:**
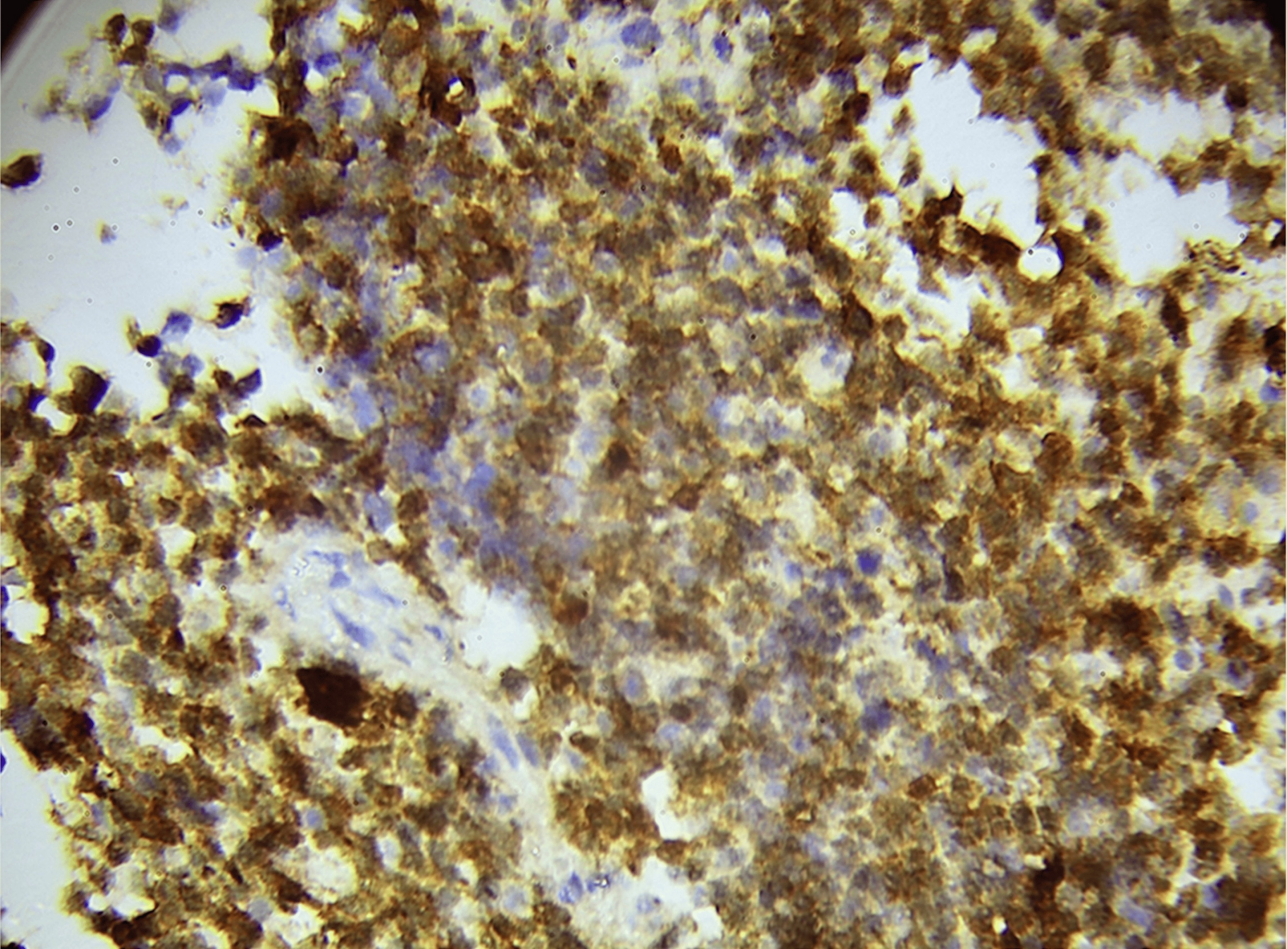
The presence of the IDH1 (R132H) mutation is confirmed by the distinct cytoplasmic and nuclear staining seen in a representative high-grade glioma section exhibiting IDH1 (R132H) positivity. IDH1: isocitrate dehydrogenase-1.

**Figure 8:**
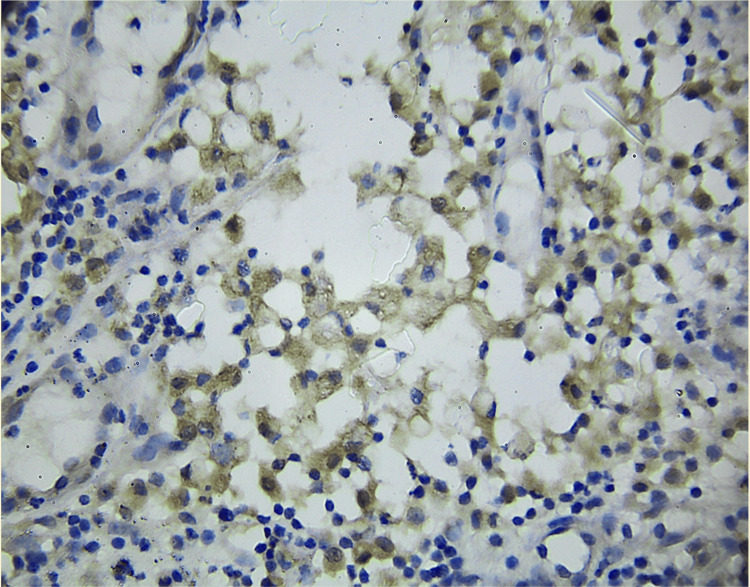
IDH1 staining of a representative low-grade glioma section shows clear, positive immunoreactivity (usually for the R132H mutation). A defining feature of IDH1-mutant status is the obvious cytoplasmic and nuclear staining seen in tumor cells. IDH1: isocitrate dehydrogenase-1.

**Figure 9:**
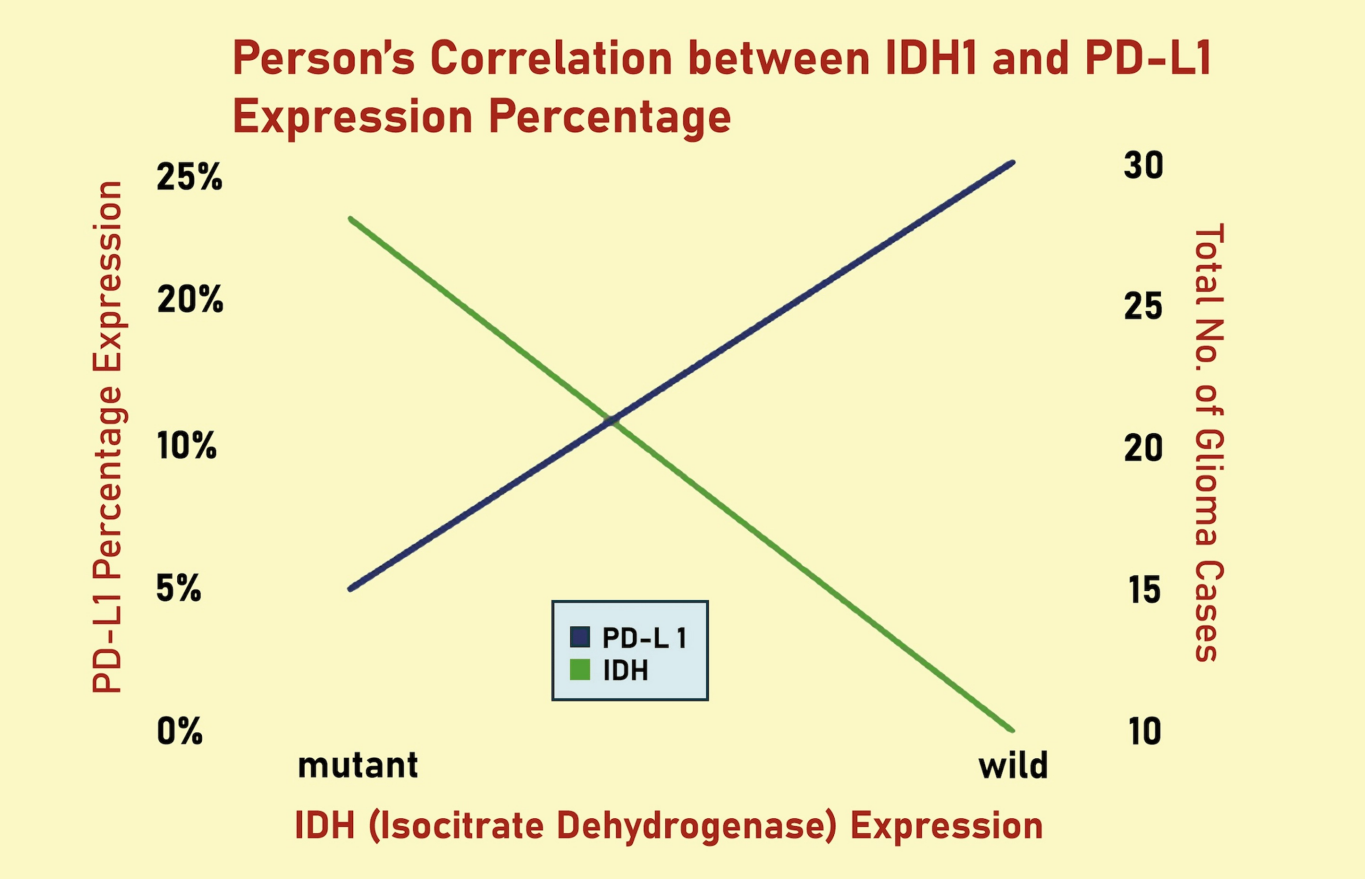
A scatter plot showing how PD-L1 and IDH1 (R132H) expression relate to one another in glioma samples. With the x-axis showing IDH1 (R132H) expression levels and the y-axis showing PD-L1 expression levels, each data point represents a distinct case. A significant inverse correlation is shown by the fitted trendline and supporting statistics (p-value and Pearson's correlation coefficient): PD-L1 expression tends to decline as IDH1 (R132H) expression rises. The statistical relationship noted in the Results section is supported by this graphic representation. PD-L1: programmed death-ligand 1; IDH1: isocitrate dehydrogenase-1.

According to the WHO classification of Central Nervous System Tumors, 5th edition, WHO grade 4 gliomas (Figures 1, 2) exhibited a higher PD-L1 positivity rate of 27 cases (75%), whereas WHO grade 2 gliomas (Figures 3, 4) demonstrated a PD-L1 expression rate of six cases (66.7%) (Figure 5). The potential of PD-L1 as a therapeutic target was highlighted by the fact that it was expressed in 33 (73.3%) glioma cases overall (Figure 6). IDH1 (R132H) expression (Figures 7, 8) and PD-L1 were found to be inversely correlated (Figure 9) (Pearson's correlation, p < 0.05).

**Figure 10:**
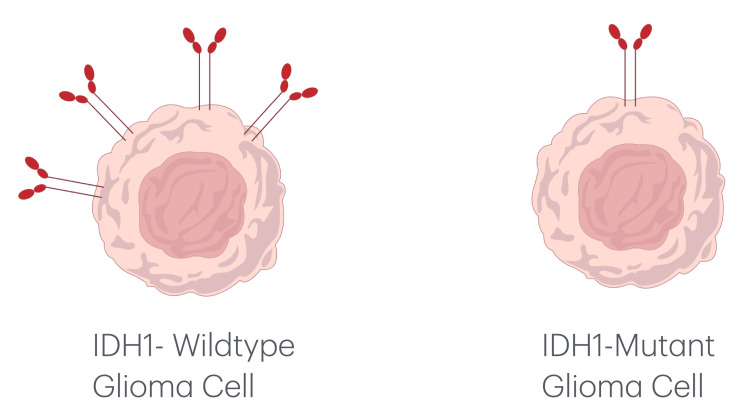
An illustration of two glioma cells showing how the presence of an IDH1 (R132H) mutation affects PD-L1 expression. The IDH1-mutant glioma cell (right) exhibits decreased PD-L1 expression, whereas the IDH1-wildtype glioma cell (left) exhibits elevated levels of PD-L1 (red membrane proteins). This disparity in surface molecule abundance could affect glioma treatment outcomes and immune evasion strategies. PD-L1: programmed death-ligand 1; IDH1: isocitrate dehydrogenase-1.

## Discussion

Reduced PD-L1 positivity was linked to higher IDH1 (R132H) expression, indicating that IDH1 (R132H) mutations may alter immune checkpoint pathways and affect tumor immune evasion strategies (Figure 10). A weakened immune response against malignant glioma cells may be suggested by decreased PD-L1 expression. On the other hand, the high expression of PD-L1 in most gliomas underscores its potential as a biomarker and therapeutic target, although its effectiveness may vary depending on the presence or absence of an IDH1 (R132H) mutation. The graphic representation (Figure 6) highlights PD-L1 positivity's potential as both a biomarker and a therapeutic target by providing a clear visualization of its prevalence across all glioma samples analyzed.

Despite advancements in surgical, radiological, and chemotherapeutic techniques [16-18], glioblastoma multiforme (GBM) remains one of the most aggressive and difficult primary brain tumors to treat, with a persistently poor prognosis [1,2]. This study aimed to explore the expression of PD-L1 and IDH1 (R132H) in gliomas to evaluate their potential as prognostic biomarkers and therapeutic targets. The results reveal significant interactions between PD-L1 and IDH1 (R132H) in the tumor microenvironment, shedding light on the immune landscape of gliomas.

Consistent with previous research demonstrating high PD-L1 expression in glioblastomas, our study found PD-L1 expressed in six (66.7%) grade 2 gliomas and 27 (75%) grade 4 gliomas [19,20]. The overall expression rate of 33 (73.3%) emphasizes the role of PD-L1 in tumor immune evasion. In pediatric glioblastoma, PD-L1 expression was detected in 20 (44%) cases [21]. These findings align with data suggesting that a more immunosuppressive tumor microenvironment, driven by PD-L1 expression, may contribute to poor prognoses in glioblastoma patients [22].

**Figure 11:**
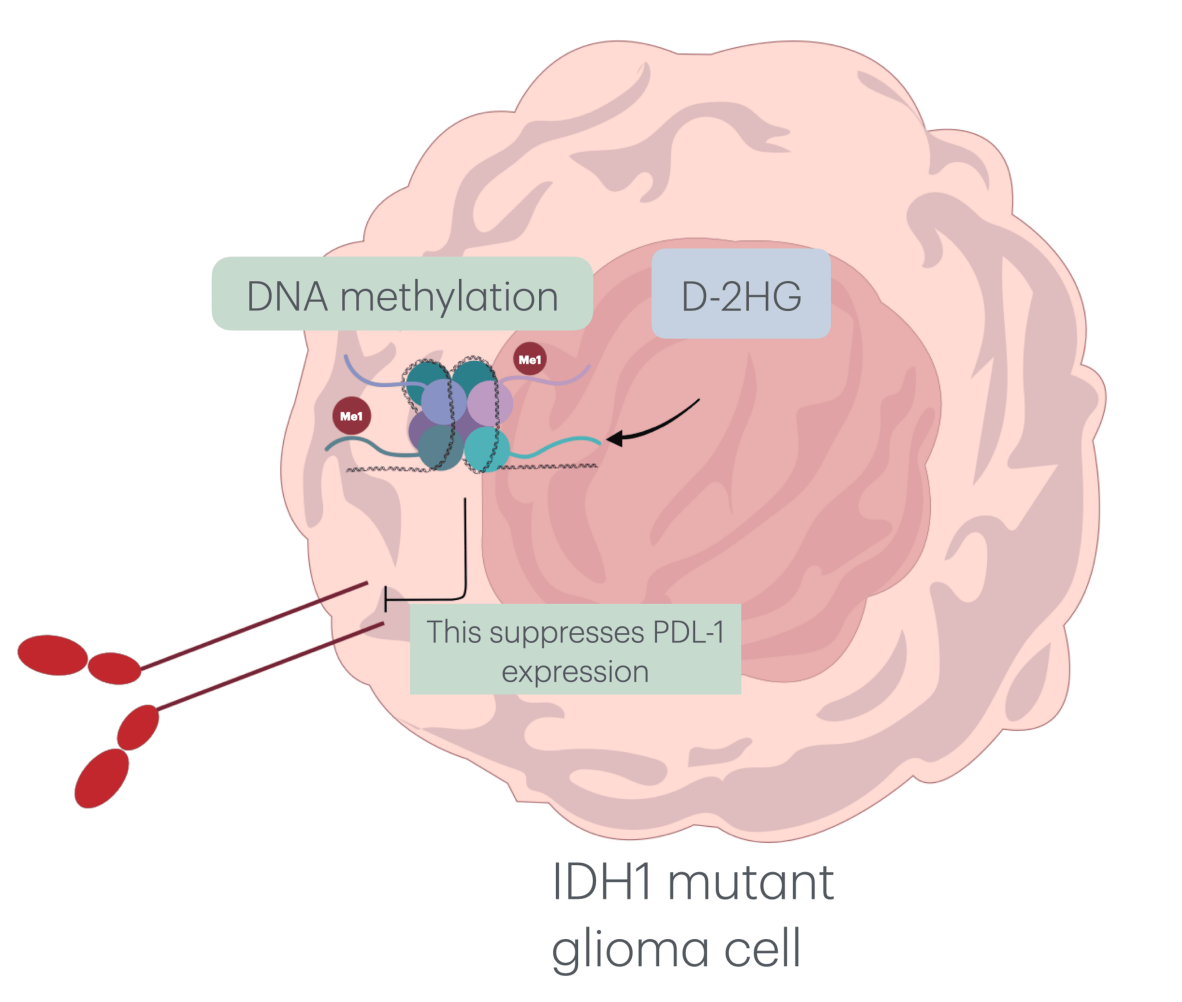
Diagram showing how glioma cell epigenetics and PD-L1 expression are affected by IDH1 mutations. The oncometabolite D-2HG, which is produced by the IDH1-mutant cell, causes DNA hypermethylation, which is indicated by methyl groups on DNA strands, and suppresses the expression of PD-L1 at the surface of tumor cells. The immune landscape of the tumor may change as a result of this epigenetic reprogramming, which could also affect how well treatments work. D-2HG: D-2-hydroxyglutarate; PD-L1: programmed death-ligand 1; IDH1: isocitrate dehydrogenase-1.

**Figure 12:**
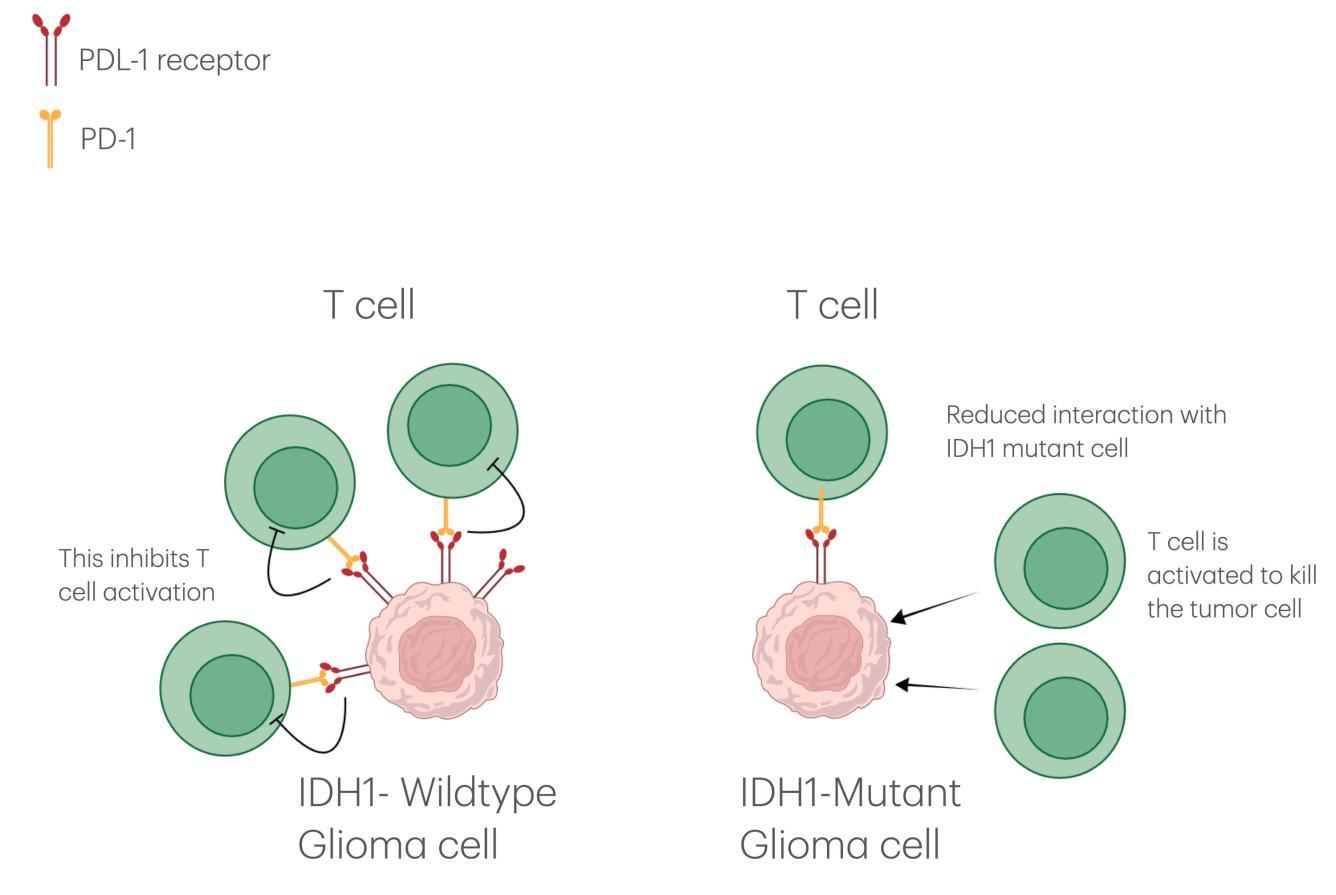
Illustration of T cell-tumor cell interactions that are influenced by the presence of IDH1 mutations and PD-L1 expression. By activating PD-1 receptors on nearby T cells, the IDH1-wildtype glioma cell (left) with elevated PD-L1 expression promotes immune suppression. The IDH1-mutant glioma cell (right), on the other hand, exhibits decreased PD-1/PD-L1 binding and lower PD-L1 levels, which may improve T cell-mediated immune responses. IDH1: isocitrate dehydrogenase-1; PD-1: programmed death-1; PD-L1: programmed death-ligand 1.

Additionally, our results indicate that IDH1 (R132H) mutations are inversely correlated with PD-L1 expression, suggesting a complex interplay between immune checkpoint regulation and metabolic pathways (Figure 11). Unlike IDH1-wildtype gliomas, IDH1 (R132H) mutations suppress PD-L1 expression through DNA hypermethylation, leading to a less immunosuppressive microenvironment (Figure 12) [23].

This finding aligns with prior studies indicating that IDH1 (R132H) mutations enhance treatment responsiveness and are associated with improved recurrence-free survival [23,24]. In contrast, IDH1-wildtype gliomas show elevated PD-L1 expression, which correlates with a more immunosuppressive tumor microenvironment and poorer clinical outcomes [25].

The high PD-L1 positivity rate in gliomas reinforces its potential as a therapeutic target. However, PD-L1 inhibitors may have reduced efficacy in IDH1 (R132H)-mutant gliomas, as suggested by the inverse correlation between IDH1 (R132H) expression and PD-L1 levels. This observation supports existing research showing that IDH1 (R132H) mutations suppress immune checkpoint pathways and alter tumor immunogenicity [25,26]. These findings highlight the need for personalized immunotherapy approaches that account for molecular subtypes, such as IDH1 (R132H) mutation status, to optimize clinical outcomes.

Further analysis reveals that mutant IDH1 (R132H) protein, although less common in high-grade tumors, may influence treatment sensitivity, epigenetic regulation, and patient prognosis (Figure 7). Lower-grade gliomas, frequently harboring IDH1 (R132H) mutations, are associated with better prognoses, improved responses to specific therapies, and distinct metabolic and epigenetic profiles (Figure 8).

Prior research has shown that the metabolic activity and epigenetic profile of gliomas, particularly in IDH1-wildtype tumors, play a significant role in regulating PD-L1 expression [23,24]. Our findings align with this hypothesis, suggesting that IDH1 (R132H) mutations influence immune checkpoint expression through epigenetic mechanisms. Moreover, the observed variation in PD-L1 expression across glioma grades underscores the need for further investigation into its prognostic value and therapeutic potential [19,20].

Understanding the interplay between PD-L1 expression, IDH1 (R132H) mutations, and the tumor microenvironment is essential for advancing glioma treatment. Integrating molecular data into clinical decision-making may guide the development of targeted immunotherapy strategies. Additionally, emerging diagnostic technologies, such as immuno-positron emission tomography (PET) imaging and machine learning-based Raman histopathology, could enhance the accuracy of PD-L1 detection and improve treatment planning [27,28].

Limitations of the study

However, several limitations should be acknowledged. The retrospective nature and small sample size of this study may limit the generalizability of the findings. Furthermore, the lack of longitudinal follow-up data restricts our understanding of the clinical impact of IDH1 (R132H) mutations and PD-L1 expression on patient outcomes. Larger prospective studies with broader molecular profiling are necessary to validate these results and further investigate the complex interactions between immune checkpoint pathways and metabolic alterations in gliomas.

## Conclusions

The high expression of PD-L1 in 33 cases (73.3%) out of 45 cases highlights the important role of this protein in gliomas and raises the possibility that it could be used as a biomarker and therapeutic target. PD-L1 and IDH1 have an inverse relationship, which emphasizes how crucial it is to take molecular subtypes into account when creating tailored immunotherapy plans.

These findings open the door for further research aimed at improving prognostic, diagnostic, and therapeutic approaches by deepening our understanding of glioma immunobiology. Patients with glioblastoma may eventually experience better clinical results and a higher quality of life with customized treatments that target both immune and metabolic pathways.
